# Breaking Up and a New Beginning When One’s Partner Goes into a Nursing Home: An Interview Study

**DOI:** 10.3390/healthcare9060672

**Published:** 2021-06-04

**Authors:** Gerd Ahlström, Nina Stååhl Markeling, Ulrika Liljenberg, Helena Rosén

**Affiliations:** 1 Department of Health Sciences, Faculty of Medicine, Lund University, 221 00 Lund, Sweden; helena.rosen@med.lu.se; 2 Home Healthcare Unit, 275 31 Sjöbo, Sweden; nina.markeling@sjobo.se; 3 Social Sector Team, 243 21 Höör, Sweden; ulrika.hansson@hoor.se

**Keywords:** spouse caregivers, next of kin, psychological distress, transition, frail elderly, multimorbidity, nursing home, elderly health care, palliative care, qualitative research

## Abstract

In aging societies worldwide, spouses take on great responsibility for care when their partner continues to live at home. Nursing home placement occurs when the partner becomes too frail due to multimorbidity, and this will cause a change in the spouse’s life. This study aimed to explore the spouse’s experience of their partner’s move to a nursing home. Two interviews were conducted at 9-month intervals within the project entitled “Implementation of Knowledge-Based Palliative Care in Nursing Homes”. Thirteen spouses from both urban and rural areas were included, with an age-range of 60–86 years (median 72). Qualitative content analysis was performed. The main findings were captured in two themes: Breaking up of close coexistence and Towards a new form of daily life. The first encompassed processing loneliness, separation and grief, exhaustion, increased burden, and a sense of guilt. The second encompassed a sense of freedom, relief, acceptance, support and comfort. Professionals in both home care and nursing home care need to develop and provide a support programme conveying knowledge of the transition process to prevent poor quality of life and depression among the spouses. Such a programme should be adaptable to individual needs and should ideally be drawn up in consultation with both partners.

## 1. Introduction

An older person’s move to a nursing home will involve a critical transition for their next of kin, affecting the well-being of the whole family [1]. The policy of “aging in place”, means that older people stay in their own homes as long as possible [2]. Here, they receive comprehensive care and assistance. Informal caregiving is much more comprehensive than personal care and often consists of emotional support, keeping the person company, checking on the person’s safety, house-work and gardening, paperwork, and taking the person out for a walk [3]. Providing care for older people can involve both positive experiences (such as enjoying companionship and being rewarded) and negative experiences (such as feeling overburdened), and the two often co-exist. The positive experiences can protect against negative, but the type of task, rather than the number of tasks, tends to be more important when it comes to how caring is perceived [4,5].

Spouses are often responsible for much of the caregiving for their partner before placement in a nursing home. The time in nursing homes, therefore, implies palliative care when the older people are severely ill due to multimorbidity [6,7]. Older adults who have been cared for at home by their spouse generally have a greater need for care when they go into a nursing home because they usually have a more serious illness than who have lived alone [8]. The increasing number of hours devoted to care is a significant predictor of care burden [9], and it is more common for men to receive informal care than women [10]. A study involving spouses highlighted that their lives were characterized by reduced individual freedom and increased responsibility for the care of their sick partner—it was as if their lives had shrunk [11]. An older person’s move to a nursing home is often triggered by the fact that their spouse can no longer cope with providing the care that they need [12,13,14]. Spouses can become increasingly aware of their condition being unteanable, though it is often home care workers, nurses, or children who first raise the question with the spouses of placement of the partner in a nursing home [15]. When a person can no longer manage to care for their partner at home, it is common for them to feel a sense of guilt and shame [16,17].

Research suggests that next of kin may be unprepared for what is involved in nursing home placement and may experience the support and commitment from nursing home care staff as insufficient or unpredictable [1]. Studies have found that many next of kin experienced ambivalent feelings. On the one hand they felt relief after the stressof providing home care for their partner, on the other hand they often had a troubled conscience about their contribution to their partner’s having to go into a nursing home. Furthermore, a sense of losing control can occur when handing over care to a nursing home [18,19]. It can be difficult for next of kin to let go, and there is often a desire that the partner should receive the same care at the nursing home as was provided in the couple’s own home. This can lead to the next of kin’s attempting to control care staff and showing a strong desire to be involved in their partner’s care [19,20,21].

During the process of a person’s move to nursing home, their spouse often undergoes a transition from one phase of life to another that involves changes of identity, abilities, and/or roles within the relationship [22,23]. A spouse who continues to live at home needs to process and incorporate the difficult decisions that have been made for their partner, find a new role, and adapt to the new situation. Research has explored the experiences of next of kin in a broad sense but few studies focus explicitly on spouses’ experiences of the transition process when their partner moves to a nursing home [15,24,25]. A healthy transition process can occur with the right support for the person who moves into the nursing home and their spouse [26]. Making the spouse’s experiences visible may help care staff to respond adequately and provide the necessary support.This being the case, the aim of the present study was to explore the spouse’s experience of their partner’s move to a nursing home.

## 2. Materials and Methods

This study used a qualitative design based on interviews with the spouses of older persons who had moved into a nursing home. The interviews were conducted in connection with the project entitled “Implementation of Knowledge-based Palliative Care” (KUPA) [27] (the acronym derives from the Swedish project title). The project is (bears the reference NCT02708498) in the Clinical Trial Register. More details about the educational intervention directed towards different professionals in nursing homes can be found in the published study protocol [27,28].

### 2.1. Setting

In Sweden, municipalities have formal responsibility for the care of older people, generally through different community care and services provided in their own home and in nursing homes or special housing [29]. The right to nursing home placement is based on an individual’s comprehensive need for care. Each person in a nursing home has a bedroom, a small kitchen, and their own bathroom. In nursing homes, older people have access to health care around-the-clock provided by a nurse. A registered nurse is in charge who instructs and delegates tasks to assistant nurses. The latter are the predominant professionals; others are occupational therapists, physiotherapists, and unit managers. Physicians in primary health (a general practitioner) work as consultants for nursing homes.

### 2.2. Sample Procedure and Participants

A purposeful sample was included, with spouses of older people living in the selected nursing homes in two counties in southern Sweden [19,30]. Both urban and rural areas were covered. The inclusion criteria were that the spouses regularly went to see the older person at the nursing home and that they could understand and speak Swedish [27]. A member of staff designated as contact person at each of the nursing homes assessed which of the next of kin fulfilled the inclusion criteria. This person gave the next of kin brief information about the study and asked whether they were interested in further contact. For those who were, the name and telephone number were given to the researcher, who contacted them by telephone, told them more about the study, and then asked them whether they wished to confirm their willingness to participate.

Eligible for this study were 13 spouses from the total group of 60 next of kin included in the KUPA project (mostly adult children). All agreed to participate in two interview,. but three people dropped out of the second interview because their wife/husband who lived in a nursing home had in the meantime died. The median age of the study group was 72 years (range 60–86), the majority were women, and three spouses had an education in healthcare. The majority went to the nursing home every day (*n* = 8), the others more than once a week (*n* = 5) (Table 1). One of the spouses in the study group moved together with the partner into the nursing home.

### 2.3. Interviews and Data Collection

The time and place for the interviews were decided according to the spouse’s wishes. The chosen places were a conference room at the nursing home and, in a few cases, their own home. Before the start of each interview, oral and written information about the study was offered and written consent was provided.

The interviews began with a broad introductory main open-ended question: “How do you experience your life-situation?” When the participant described the situation concerning the move to the nursing home, follow-up questions were tailored to the individual and designed to elicit depth and breadth. Common follow-up questions were: “How did you feel about the move?” and “Do you want to tell more about…?”

The participants spoke candidly about the changes and their feelings about their partner’s move to the nursing home. The interviewer facilitated their narration through active listening and by probing follow-up questions. The participants were given time for reflection during the interviews. The interviews lasted 45–90 min, were digitally recorded, and were transcribed verbatim before analysis. They were conducted in the month before the KUPA project educational intervention started. Ten of the 13 spouses were re-interviewed after the intervention 9 months later, with the same interview guide. Thus, 23 interviews were included in the analysis. The time between the interviews was dependent on the length of the intervention ongoing for the staff in the nursing homes. Descriptions of participants’ partners’ placement in nursing home and their own changed life-situations emerged in both interviews.

### 2.4. Data Analysis

Qualitative content analysis can be applied to text generated through different data collection methods, including interviews [31,32,33]. First, the 23 verbatim interview transcripts were read through several times by all authors to acquire a greater understanding of the text as a whole. The next steps were performed by the second and third authors, which started with identifying meaning units relevant to the aim of the study. The meaning units [33] varied from a few sentences to a paragraph and were later condensed into shorter units to bring their essential content into prominence (Table 2). The next step was creating codes, which were then categoried. The categories with common codes were grouped and in the end two themes emerged from the interpretation of the latent content. This occurred through repeated moving back and forth between consideration of the manifest content, codes and categories as well as consideration of the data as a whole. The categories had more manifest content, whereas the themes were based on the interpretation of the latent content [33]. Next, the first and last authors reviewed the steps of the analysis. Then there were several discussions which led to some changes being made in the formulations, until a consensus between all the authors was reached (Table 2) [34].

## 3. Results

Two themes emerged from the analysis: Breaking up of close coexistence and Towards a new form of daily life. The themes consisted of 5 and 4 categories respectively (see Table 3).

### 3.1. Breaking Up of Close Coexistence

The breaking up of close coexistence (Table 3) extended from before to after the partner’s move to a nursing home, and the processing of emotions moved back and forth after the placement but was less dominant. Spouses were saddened to see that their partner’s health was worsening, which gave rise to a sense of separation. They at first struggled on together at home, although with a greater need for care and attendance in the case of the partner and a greater burden on the spouse with regard to practical tasks that could no longer be shared. Symptoms of fatigue and exhaustion increased despite the contribution of other family members and home-help services. Whether consciously or sub-consciously, spouses were constantly aware that there would come a point where their partner would have to go into a nursing home. This generated a sense of guilt about no longer being able to cope with the situation, letting the other person down, and not being true to the marriage vow concerning “in sickness and in health”.

The spouse’s sense of separation became more acute after the partner’s move, and was especially felt at the end of each visit to the nursing home. Grief sapped the spouse’s energy, although this grief diminished in strength with the passing of time. There was a feeling of loneliness despite being one of a couple, fuelled by the awareness of now having to make all decisions on one’s own. After the move, spouses also felt greater physical loneliness: the marital home seemed empty and silent, and factors such as clothes and an empty bed in the bedroom acted as reminders of what had been lost. Life became stressful and there was a loss in well-being and satisfaction.

#### 3.1.1. Loneliness

Before the move, spouses described some feelings of loneliness associated with being tied to the home and experiencing a narrowing of social networks as their partner required more attendance. There was also a feeling of loneliness in having to bear all responsibility for making major decisions.

*I feel lonely, awfully lonely. (Spouse 6, interview 2)*

Most spouses felt lonely after their partner went into a nursing home, and several found it difficult to be at home because that was when the loneliness was worst. They missed the intimacy and having someone to talk to, and noted that there was “such a silence.” The loneliness was mostly present in everyday life for those who had lived together for many years. Even when sitting and watching TV, the spouses missed having their partner close to them. One spouse said that their loneliness got worse as time passed.

*It’s the loneliness that’s worst. I might just as well be here [at the nursing home] as sitting on my own at home. (Spouse 11, interview 2)*

Some spouses said that their children did not come and see them as often now, or did not come at all but just went to the nursing home instead. This was a further source of loneliness. Visiting the nursing home was described as a way to ease the loneliness and for some spouses, and contact with staff was an important part of this. Filling the day with activities such as gardening and going to see grandchildren was a help, as was having a pet.

*There’s nothing worse than sitting on your own at home, so I’ve got myself two dogs. (Spouse 11, interview 1)*

#### 3.1.2. Separation and Grief

Spouses experienced grief over the separation and not being able to live together any longer, especially as there were many memories from their long relationship that painfully brought home how things had changed. They also expressed some anger over the way things had turned out and over the finality of the separation.

*Things seem to be going well anyway, even though I still want to have him home. Yes, that’s what I want deep down, there’s no getting away from it. (Spouse 9, interview 2)*

It was difficult for the spouse leaving their partner in a nursing home. It did not appear to make a difference knowing that it was a good nursing home; there was always a dream of the partner returning home. Spouses described experiencing grief because aging had not turned out as expected, and because of the turn that life had taken. There was plenty to think about “Why did things turn out this way?”, “Did I do the right thing?” In one case, the nursing home placement was prompted by a physician saying “You’ve got to take care of yourself,” but the spouse reported that everything went so fast you could not really keep up. The sense of separation was especially acute at the end of a visit to the nursing home.

*We’ve lived together for a lot of years, 60 it is now, and it’s so difficult to leave her each time. (Spouse 7, interview 1)*

One wife said that she had been going to sort out and throw away her husband’s things that were still in the marital home, but in the end could not face it. It was as if time stood still. For several spouses, the grieving process went on for a long time and was hard to accept.

#### 3.1.3. Exhaustion

The time leading up to the move was often physically and mentally exhausting for the spouses. There was the burden of worrying about the future as well as the increased burden of care. Despite home-help services and care, and in some cases relief housing, spouses’ situation had become unsustainable. They were sapped of energy and could not carry on.

*I couldn’t go on like that, not being able to sleep at night. In the end I got irritated. (Spouse 12, interview 1)*

*During the night I’d lie waiting, worrying, unable to relax, trying to hear whether she was up. I got no sleep and nothing was going right. (Spouse 7, interview 1)*

The spouses described being worn down by the need to provide constant attention and the unceasing worry that something bad was going to happen. Many had reached their limit before asking for external assistance, and this assistance seemed to have come too late. Events in everyday life were experienced as stressful and there was a sense of insecurity in one’s own home. The spouse’s worry became even greater when they perceived that their partner’s condition was becoming so bad that more care was required than could be provided at home.

*It had to be a nursing home, I’d waited far too long. I ought to have asked for help much sooner, I can see that now. I was exhausted and on the sick list. (Spouse 8, interview 1)*

#### 3.1.4. Increased Burden

The practical burden for spouses increased in the period leading up to the move and after the move. They had no one to help when it came to such things as paying bills, cleaning the house, and seeing to the garden. Furthermore, there were tasks that had been the sole responsibility of the partner who now lived in the nursing home, and spouses had to learn these tasks from the beginning. The burden was often especially heavy during the period immediately before and after the move. Many practical decisions had to be made.

*There are papers coming all the time, lots of decisions to be made. What’s to be done about the summer cottage and what needs doing in the flat? There’s a lot to think about. (Spouse 6, interview 2)*

#### 3.1.5. Sense of Guilt

Spouses reported often feeling a sense of guilt. In some cases this was related to not being able to cope any longer, not having the energy, or giving up. For example, some spouses knew that their partner would be better off at home but were unable to handle it. The sense of guilt was greater if the partner did not like being in the nursing home, but realized that there would be no going back.

*That’s the worst part, feeling I can’t handle it. (Spouse 12, interview 1)*

They were disappointed at not being able to live up to their image of how “a good spouse” should behave. There was guilt if they did something else instead of going to see their partner, and guilt if the partner expressed disappointment and anger about not being able to live at home any longer. They found it hard being confronted with the fact that their partner was unhappy. Spouses reported that one way of mitigating the sense of guilt was to remind themselves that the situation before the move had become unsustainable.

*(Sighs) I feel that I betray him in some way when he does not come home for real. But I know It’s not possible. I know it, and yet it feels awful. (Spouse 4, interview 1)*

### 3.2. Towards a New Form of Daily Life

Towards a new form of daily life (Table 3) is the second theme, which principally concerned the period following the move when things seemed more positive and hopeful. A sort of peace emerged from spouses’ acceptance of their new situation; relaxation derived from the gradual recognition that the decision that their partner should go into a nursing home was right in the circumstances. Their partner’s daily life in the nursing home was better than before, and so was their own life. They reported a sense of freedom and relief as a result of not having to bear the responsibility for their partner’s health and welfare any longer. Having one’s partner go into a nursing home was described as a bewildering experience, but in the course of time the grief and the sense of separation gave way to an improved quality of life. Spouses reported that the nursing home offered support and comfort, and they could embrace a new, well-functioning form of daily life.

#### 3.2.1. Sense of Freedom

The period closely following the move brought a sense of freedom to spouses remaining at home, despite the sense of guilt surrounding the difficult decision. While life had previously been dominated by attending to the care needs of their partner, this was no longer the case, even though spouses’ thoughts were still with their partner.

*I’ve got another sort of freedom now, of course. I can arrange my day as I want. Before, it was all so regimented and I was tied to the home 24 h a day. (Spouse 5, interview 2)*

After their partners had gone into a nursing home, spouses had more chance to pursue their own interests. There was an enormous sense of freedom in being able to go out without being worried by thoughts of what might be happening at home.

*Now I can get drive over and see the children whenever I like, so in that way things have got better. I’m free to do what I want now. (Spouse 12, interview 1)*

#### 3.2.2. Relief

Spouses expected that there would be a long wait before getting a small flat in the nursing home. When this was not the case, they were surprised and experienced significant relief. It was a relief to feel that the nursing home was providing their partner with the requisite care and attention. They trusted in the care and felt free from worry about their partner. Now there was a chance to relax.

*It was liberating to be relieved of the burden of being so tied day and night. (Spouse 5, interview 2)*

The move to the nursing home considerably reduced the pressure on the spouses that remained at home. They were grateful that the pressure had diminished and that they felt better, both mentally and physically. It was a relief not having practical care tasks to perform, not having all of the responsibility, and not being tied to caregiving 24 h a day. It made such a difference having the nursing home staff take over, especially as it had been difficult (sometimes impossible) to relax before. When they saw that their partner appreciated their visits, it gave a sense of having a task, which facilitated the situation.

*It is good that we are living so close and that I can visit her and it is probably*

*security for her when I come there too. I go there every day, and it is a relief*

*also for the staff because I can help. (Spouse 1, interview 1)*

#### 3.2.3. Acceptance

Spouses did not want their partner to move but felt that there was not much they could do when there was no hope of better health for their partner. They tried to cope with living in the present and not looking forward or back. They spoke of a process of the gradual acceptance of their changed practical circumstances and their feeling of the loneliness. Their coping with their changed life-situation was facilitated when the partner who lived in a nursing home was glad and appreciated their visits. The change had been considerable, and they were proud of having come to terms with it.

*I’ve had to do most things myself recently, in the house and everything. I’ve had to do the things he used to do. I used to be scared to death of windowed envelopes but not anymore. (Spouse 12, interview 1)*

Spouses also reported searching for new strategies and new sources of enjoyment. They had different ways of learning to accept their changed situation. One wife said that she never went into the living-room any more, to protect herself from being overwhelmed by the grief she bore every day. New routines had to be developed, and for some spouses the visit to the nursing home had become an important part of their day.

*Before, I thought it was sad to drive home from the nursing home because I thought it would not be better. I still believe in that way, but try to push it away from me, it will not be better if I dig down into it. (Spouse 13, interview 2)*

#### 3.2.4. Support and Comfort

Family was a source of comfort for spouses. Feeling welcome among one’s family and receiving support from them was an important part of accepting the decision that one’s partner should move to a nursing home. Spouses appreciated the communication with their children and friends in making the decision that their partner’s move was best for them both. When the partner had gone into a nursing home life had become more secure, offering a better future for both.

*He seems to be content, I can’t see anything negative. I’ve got confidence in them. I’m grateful, very grateful. (Spouse 8, interview 2)*

*I can’t complain, my husband couldn’t be in a better place. He’s where he’s best off, he’s lucky it turned out so well, and so am I. (Spouse 9, interview 2)*

Visits to the nursing home were a source of comfort, and spouses were grateful for the support of staff. The nursing home atmosphere was reported to be welcoming and they found it was good to encounter other next of kin in the same situation with whom they could share experiences, thoughts and feelings. Several spouses expressed appreciation for the arranged meetings for next of kin.

*The meetings for us next of kin help you cope. I mean, the people I meet there have been in the same situation. It feels good being at one of those meetings. (Spouse 11, interview 1)*

The spouse that had moved into the nursing home with their partner appreciated the opportunity to continue to live together and appreciated that the environment was like home, with kindly staff that helped the partner with medicine and hygiene. There were also more things to do than had been available previously, such as listening to singers and going to the seniors’ shop.

## 4. Discussion

The findings in this study highlighted spouses’ experiences before and after their partner went into a nursing home as involving a process that could both positively and negatively affect their well-being. Spouses experienced loneliness, separation and grief, exhaustion, and increased burden in everyday life as their partner’s health deteriorated. They felt guilty when placement at a nursing home became inevitable, and became the only solution. This sense of guilt had to be handled but was not easy to accept, even though they understood that they could not manage the care situation any longer. Our findings also showed positive experiences after the move, such as sense of freedom, relief, acceptance, support, and comfort when they accepted the decision and started to look forward to a new phase in life. The emotions experienced before the move remained after it, but tended to be less dominant andless persistent.

The themes found in this study, Breaking up of close coexistence and Towards a new form of daily life, can be understood from the perspective of transition theory, in terms of the separations, processing and incorporating phases [35]. Spouses in our study experienced the transition phases as back and forth movement over a period of time rather than as a straight linear process, which is consistent with transition theory [35]. This back and forth movement was found before by Paterson [36], who also emphasizes that change processes are not linear regardless of type of transition. According to Meleis, transition is a central concept involving change and development as well as continuity and interruption [35,37]. The concept of transition also entails personal change and adaptation, including reconstruction of a valued self-identity. The duration of the processing phase depends on why the transition was necessary and how it is was carried out [23].

### 4.1. Breaking Up of Close Coexistence

This theme included the separation phase and parts of the processing phase of transition theory [35]. The loneliness was expressed in spouses’ increased sense of isolation when their social networks shrank, and the personal closeness to and dialogue with their partner were no longer the same as before the move. Førsund and colleagues [38] also found an intense feeling of loneliness among remaining spouses, almost like a physical feeling of absence. Larsson [39] highlighted the difficulties of finding a new context, whereby many spouses had a desire to experience togetherness but lacked the emotional strength to seek new contacts. Our findings revealed that visits to the nursing home eased spouses’ loneliness. This was consistent with the findings of Førsund and colleagues [38], who reported that relationships characterized by respect and love were maintained by frequent visits to the partner at the nursing home. Awareness of the significance of spouses’ visiting their partner is important when it comes to care staff’s ability to provide person-centred care.

Spouses felt separation and grief and felt they were in between an old and a new way of life, in a no-man’s-land [40], confused and alienated. A typical attempt to move on in this part of the process was sorting out and throwing away the partner’s things, while at the same time not being able to cope with emotions such as a sense of separation and grief. It was as if time stood still for spouses when they were in both the processing and separation phase at the same time. Burton and colleagues [41] found it was also common for elements from one or more transition phases to be experienced in parallel, such as changes in the caregiving role and the consequences of their partner’s moving into a nursing home.

The spouses in our study experienced grief and a feeling of separation in conjunction with their partner’s having to move to a nursing home because of worsened health. According to our results, when the process of separation and grief went on, this was expressed as anger over the way things had turned out and the finality of the separation. Transition theory indicates that questions about the meaning of life arise during the separation phase. In our findings, spouses expressed the experience of loss over not being able to share everyday life as sadness that the future was not going to be as they had hoped and expected. The losses that arise from a separation may lead to a range of consequences, such as loss of strength and energy [42]. The transition interfered with everyday life, and in the first phase, often resulted in distress and uncertainty. One spouse spoke of this as having to adapt by “navigating in unknown waters.” They were grieved that they could not take care of their partner at home and have the situation as it was before, even though they knew this was not possible. At that stage of the transition, it was evident that spouses had not accepted their new situation.

Spouses described the time before the partner’s move to the nursing home as stressful, and noted they became increasingly exhausted. Despite home-help services and care, the situation became unsustainable. This, together with an increased burden, led spouses to realize that their partner could not stay at home any more, which was identified as a predictor of care burden by Kim et al. [9]. Increased burden was described by spouses in terms of putting a lot of energy into getting their partner organized for the nursing home and then not having the strength left to take care of their own lives. There was also concern about their future, including about whether they should stay in their current accommodation or move to something smaller. The burdens in everyday life also included new tasks that had to be performed, such as going through the partner’s papers. It is well-known that informal caregivers heavy high emotional and practical burdens, which is why the municipality is obliged to inform and give individualized support to prevent exhaustion [11].

The category “sense of guilt” that emerged in our results was attributed to not being able to cope any longer as the partner deteriorated before the move to nursing home. Spouses felt guilty when they did something else instead of going to see their partner at the nursing home. This feeling became even worse when their partner was unhappy about not being able to live at home any longer. Our finding is confirmed by Hennings, Froggatt and Payne [43], who reported that feelings of guilt associated with nursing home placement endured a long time after the move.

### 4.2. Towards a New Form of Daily Life

The spouses in our study reported experiencing feelings of a more positive nature when they had accepted the decision that their partner should move to a nursing home, and the move had taken place. They had taken a step “Towards a new form of daily life”. Positive feelings of freedom and relief often replaced the feelings of guilt and anxiety about no longer being able to live together with their partner. They were in a new situation and they compared this situation with their previous one. Spouses felt gratitude towards the nursing home and staff since they had confidence in the care given to their partner. In our study, transition phases experienced as parallel [41] was apparent when spouses were starting to reach the incorporation phase. New opportunities emerged, both practical and emotional, and new routines and joys in everyday life were developed and anchored. An internal reorientation had taken place, which has been described by Kralik and colleagues [22] as a fresh start where they could look at their reality with other eyes. Undergoing transitions takes time, with the length of time differing from one person to another. Most spouses in this study were through the transition process but some were still moving between the processing and incorporation phases. Transition involves different processes that last a certain time and have a dimension of movement and flow, as opposed to change, which is defined as something that happens more abruptly [35,37].

A recurring thread in the interviews was that spouses spoke of how difficult it was to come to terms with being away from their partner. Brown and Bond [44] assessed both the effect of transition on well-being and whether the reason for transition had an impact on outcomes. Transitioning from the caregiver role lifted a burden and led to improved well-being. These authors found that the act of transitioning itself had the greatest impact on well-being, rather than the reason for the transition. During transition, there can be reactions such as anxiety and confusion but also excitement and joy. Changes in roles within the family and changes in self-perception are common [22].

During a transition, there comes a turning-point when life reaches a new stage. In a completed transition, the person that has undergone the transformation will not return to their previous state [22]. Reaching a turning-point can mean a different self-understanding; it may result in new friendships, new interests or a new hobby [45]. Our participants moved between phases but also experienced grief, probably of a long-standing nature. On the surface, they could handle the situation, but at the next moment the grief was accentuated when the spouse was confronted with something that worked as a trigger. From the perspective of the theory of chronic sorrow, it is a part of the normal grieving process that happens in crucial situations and for a lot of people during a lifetime [46,47,48]. Chronic sorrow has to do with having lost something personally valuable. In our study, this was the togetherness with a loved partner. Permanent grief that is mostly managed in everyday life can become overwhelming with strong emotions at triggers [46,47,48]. In our study, reported triggers included entering the couple’s previously shared bedroom or living-room. It is essential for all professionals to understand the nature of permanent grief, and to identify whether the person uses coping strategies that lead to well-being or depression [47,48].

In our study, support and comfort from staff were essential to spouses’ well-being. Benzein and colleagues [49] demonstrated that good dialogue between care staff and next of kin promoted health and reduced suffering. This is consistent with previous research that showed that the experience of next of kin was more favourable if the nursing home offered an enjoyable and meaningful everyday life for residents, and the care staff showed commitment [50,51]. Undergoing a transition involves uncertainty, and the adaptation to the transition depends on the degree to which the person is engaged, aware, and trying to undergo it in the most creative way possible [35]. Spouses may also lack well-being, and in the worst case may experience alienation and a sense of losing control when their social and personal identities change [22,39]. Whether the result of the transition is healthy is determined to some extent by the individual’s level of knowledge. Being prepared and having relevant knowledge facilitates the transition experience [35].

One consequence of there being an aging population is that many spouses experience an acute sense of separation when their partner goes into a nursing home, which can lead to a decreased quality of life and depression. Therefore, it is important that professionals should support spouses by developing and applying support programmes. Giving the spouse the opportunity to reflect on their situation (fears, hopes, etc.) individually and in support groups can facilitate the transition process. By helping them to identify and come to terms with changes, staff in home care and nursing home care can help them see opportunities in their new life [22]. It is necessary to see the couple as a unit, which means that also the partner should be invited to participate in the support programme (for the partner, too, is undergoing a transition).

### 4.3. Methodological Considerations

Some methodological strengths and weaknesses regarding trustworthiness need to be considered when interpreting the results of this study. A strength of the study is that the participants were recruited from two counties and both urban and rural areas, which increased the credibility and transferability of the results. However, a limitation is that the number of spouses participating was no more than 13, this out of 60 who were selected for the implementation project [27]. A possible explanation is that many of the spouses are vulnerable in having their own illness or disability and that for this reason staff did not want to invite them to participate [52]. From our experience of qualitative studies we know that older people are generous in sharing their experiences if they feel they have enough energy to complete an interview. A further limitation was that we did not know how many spouses refused participation of those who were invited.

The implementation project was in the form of work-site education concerning palliative care for different professionals in 30 nursing homes, two hours once a month for six months [27]. The spouses were not involved in this education, instead, they were the ones who were to receive the benefit of the palliative care for their partner. The 9-month interval between the interviews in this study was decided upon with a view to evaluating the staff education. However, a comparison of the second interviews with the first revealed no influence of the education on the way the spouses experienced their transition. It is possible that the spouses would have appeared as being in a more critical emotional state at the time of the second interview if the interval between the interviews had been shorter. From a research-ethical perspective older spouses need to be perceived as a vulnerable group when they are in the phase of separation from their partner they have lived together with for a long time [52]. The present study was designed in such a way as to avoid laying a further burden on already vulnerable spouses. Had the interval between the interviews been longer than 9 months there would have been an more expected drop-out of spouses owing to the death of the partner. One third of seniors die within 6 months of going into a nursing home in Sweden, and this time is decreasing because of the increased home care in line with the policy of ”aging in place” [7].

The credibility of the results [53,54] is strengthened by the fact that the 13 interviewees were all spouses who had lived with their partner for a long period before the move to the nursing home. The richness of data is more dependent on partcipants’ having experience of the phenomenon than on the number of participants [34]. Their partner’s move to a nursing home was expected to have a similar meaning for the interviewees in terms of being a critical event in a couple’s life. The study design with two interviews was expected to increase the dependability of the data. This was supported by the fact that the spouses’ narratives about their partner’s move to a nursing home placement were comprehensive in both interviews [25]. Based on spouses’ answers, probing follow-up questions were asked about the move in the open-ended interviews. Both interviews were conducted after the partner had gone into a nursing home, which means that there was retrospective narration concerning different periods. The weakness with retrospective data is that memory changes. On the other hand, research has shown that strong experiences are retained longer in people’s memories [55].

One spouse (85 years old) with own illness moved to the nursing home together with the partner. The meaning units associated with this person were mostly brought together under the theme Towards a new form of daily life. This spouse’s narrative raises the question of well-being when the couple move into a nursing home together—a question which it would be interesting to study in future research. The other spouses have meaning units in most of the categories and both of the themes, though with variation.

The method applied was a systematic analytical procedure, from inductive manifest content analysis of text in the meaning units to interpretation within the framework of two descriptive themes [33,34]. Though there was focus on the aim of the study, an open mind was kept throughout the analytical procedure since it was deemed important that the spouses’ voices should be heard without influence from the researchers’ pre-understanding. Four researchers representing a mix of clinical and rsearch competence in elderly care took part in the analysis in order to increase the dependability of the results [34]. All read the interviews, and the whole text served as a point of reference for reviewing the meaning units, codes, categories and themes. Finally, to make the results more transparent, quotations from different spouses were included in the Results section. However, formulating codes, categories, and themes, as well as choosing what to quote, is always a challenge despite the careful procedure [34].

## 5. Conclusions

The findings of this study show that caregiving spouses undergo a pervasive transition when a partner needs comprehensive care and has to go into a nursing home. The early phase is marked by loneliness, a sense of separation, and grief. The separation phase continues in parallel with the next phase of moving on and coming to incorporate a new life-situation after the partner’s placement. To facilitate a healthy transition, professionals in both home care and nursing home care need to develop and provide a support programme involving knowledge of the transition process in order to prevent poor quality of life and depression among the spouses. This programme will have the best effect if it is flexible and tailor-made for spouses and at the same time offers an opportunity for the partner to participate, individually and/or in a group, online and/or face-to-face, in accordance with each person’s need of support.

## Figures and Tables

**Table 1:** Characteristics of the interviewed spouses (*n* = 13).

Background Variables	*n* (%)
Sex	
Men	4 (31)
Women	9 (69)
Pension	8 (61.5)
Employment ^1^	5 (38.5)
Highest educational level	
Elementary school	7 (53.8)
Secondary school	2 (15.4)
Trade school	2 (15.4)
University/college	2 (15.4)
Frequency of visiting the partner	
Every day	8 (61.5)
Weekly or more often	5 (38.5)

^1^ One participant worked part-time.

**Table 2:** Examples illustrating the analytical procedure.

Meaning Units	Condensed Meaning Units	Codes	Categories	Themes
**KAX1266:** [*sadly*] But during that time it was like being in a fog. *I: It’s understandable that it was hard to cope. But things got better afterwards, maybe?* Oh yes, they did, gradually. Things do get better when you’ve had time to adjust—but it was a big adjustment. We’d lived together for a lot of years—60. Then during that first time it was as if everything stood still.	During the first period it was as if everything stood still. It was like being in a fog, to have to live apart after 60 years.	Sense of unreality caused by grief	Separation and grief	*Breaking up of close coexistence*
**KAX1345:** Tough [sighs]. I felt so inadequate, somehow [breathes heavily], I really did. *I: Inadequate? In what way?* Well, with regard to *him*—not being able to take care of him [takes a deep breath]. Bad conscience. *I: What gave you a bad conscience?* Not being able to have him at home, that was the worst of all. I kept thinking about whether it would have been better for hi if he’d been at home. *I: Mm. Did he himself want to move?* No. I mean, there was no knowing, because he couldn’t speak any more. Well, he could say a weak yes or no, it’s just that it wasn’t always right anyway. I think, too, he was quite simply angry with me for not being able to cope with having him at home.	Has felt inadequate and has a bad conscience about not being able to take care of her partner.	Sense of inadequacy and bad conscience	Sense of guilt	*Breaking up of close coexistence*
**SAN2956:** Of course it took me quite some time to really accept it. And of course you feel guilty when you see that your partner isn’t happy. It’s a heavy burden, naturally. But, you see, I’ve overcome that sense of guilt, because I’ve come to realise that I really haven’t any choice.	It’s burdensome to see that your partner isn’t happy. But you can overcome the sense of guilt once you realise that you’ve got no choice.	Sense of guilt initially despite lack of choice	Sense of guilt	*Breaking up of close coexistence*
**KAD2489:** Now, afterwards, I’ve got a new freedom, I can arrange my day as I want. Before, I made sure every morning that everything was OK at home then I went and did water aerobics, which was at seven o’clock. The home-help person came every morning at eight, and I got home just a few minutes after. Same times day after day, and whatever I did had to fit in. Now I’ve got a whole new freedom. *I: Mm. A lot more chance to act spontaneously, you mean?* Yes, yes, definitely. It’s so much improved, I’ve got another range of opportunities altogether. *I: You can pursue your own interests…*	Has a sense of freedom now. There was always a routine to follow before but now things can be arranged as desired.	Sense of freedom and control of own life	Sense of freedom	*Towards a new form of daily life*

**Table 3:** Results of the analysis of themes and categories.

Themes	Categories
Breaking up of close coexistence	Loneliness
	Separation and grief
	Exhaustion Increased burden Sense of guilt
Towards a new form of daily life	Sense of freedom
	Relief Acceptance
	Support and comfort

## Data Availability

The data in the present study contain information on a vulnerable group (i.e., older spouses of older people living in nursing homes). Even though the interview text was anonymized, it includes sufficient details to potentially enable the identification of a single individual. Therefore, to approve the study, the Regional Ethical Review Board in Lund made restrictions regarding access to the data. Requests for access to data can be made by contacting the project manager (G.A.), who will consult the research ethics committee before permitting access to the data.
